# Overexpression of Thioredoxin-1 Blocks Morphine-Induced Conditioned Place Preference Through Regulating the Interaction of γ-Aminobutyric Acid and Dopamine Systems

**DOI:** 10.3389/fneur.2018.00309

**Published:** 2018-05-02

**Authors:** Xiang Li, Mengbing Huang, Lihua Yang, Ningning Guo, Xiaoyan Yang, Zhimin Zhang, Ming Bai, Lu Ge, Xiaoshuang Zhou, Ye Li, Jie Bai

**Affiliations:** ^1^Faculty of Environmental Science and Engineering, Kunming University of Science and Technology, Kunming, China; ^2^Laboratory of Molecular Neurobiology, Medical Faculty, Kunming University of Science and Technology, Kunming, China

**Keywords:** thioredoxin-1, morphine, ventral tegmental area, nucleus accumbens, conditioned place preference

## Abstract

Morphine is one kind of opioid, which is currently the most effective widely utilized pain relieving pharmaceutical. Long-term administration of morphine leads to dependence and addiction. Thioredoxin-1 (Trx-1) is an important redox regulating protein and works as a neurotrophic cofactor. Our previous study showed that geranylgeranylaceton, an inducer of Trx-1 protected mice from rewarding effects induced by morphine. However, whether overexpression of Trx-1 can block morphine-induced conditioned place preference (CPP) in mice is still unknown. In this study, we first examined whether overexpression of Trx-1 affects the CPP after morphine training and further examined the dopamine (DA) and γ-aminobutyric acid (GABA) systems involved in rewarding effects. Our results showed that morphine-induced CPP was blocked in Trx-1 overexpression transgenic (TG) mice. Trx-1 expression was induced by morphine in the ventral tegmental area (VTA) and nucleus accumbens (NAc) in wild-type (WT) mice, which was not induced in Trx-1 TG mice. The DA level and expressions of tyrosine hydroxylase (TH) and D1 were induced by morphine in WT mice, which were not induced in Trx-1 TG mice. The GABA level and expression of GABA_B_R were decreased by morphine, which were restored in Trx-1 TG mice. Therefore, Trx-1 may play a role in blocking CPP induced by morphine through regulating the expressions of D1, TH, and GABA_B_R in the VTA and NAc.

## Introduction

Morphine is the most effective pain relieving pharmaceutical, which repeated use can lead to dependence and addiction. Morphine induces addiction through stimulating dopaminergic neurons in the ventral tegmental area (VTA) (1–3). The activity of the dopaminergic neurons in the VTA is involved in the rewarding effects induced by morphine (4). Morphine first targets γ-aminobutyric acid (GABA) neurons, which results in activation of dopaminergic neurons, then leads to rewarding effects (5). Thus, the rewarding effects are regulated by γ-aminobutyric acid and dopamine (DA) systems in the VTA, which projects to the nucleus accumbens (NAc).

Thioredoxin-1 (Trx-1) has various biological activities, such as regulating redox, activating transcription factors and protecting mice from Parkinson’s disease (6–8). Nerve growth factor induces Trx-1 expression *via* activation of the extracellular signal-regulated kinase (ERK) and cAMP-response element binding protein (CREB) (6). Previous studies showed that morphine-induced Trx-1 expression *in vitro* and *in vivo* (9, 10). Geranylgeranylaceton, an inducer of Trx-1, protects mice from rewarding effects induced by morphine (10). Trx-1 overexpressing transgenic (TG) mice resisted the rewarding effects induced by methamphetamine (11). However, whether Trx-1 TG mice resist the rewarding effects induced by morphine is still unknown.

In this study, we examined conditioned place preference (CPP) in both wild-type (WT) mice and Trx-1 TG mice after morphine conditioned training and detected the levels of DA and GABA and the expressions of tyrosine hydroxylase (TH), D1, and GABA receptor B (GABA_B_R) in the VTA and NAc.

## Animals and Methods

### Reagents

Morphine hydrochloride was obtained from Shenyang First Pharmaceutical Factory, Northeast Pharmaceutical Group Corp. (Shenyang, China). Anti-mouse Trx-1 rabbit polyclonal antibody (14999-1-AP; 1:1,000) was purchased from ProteinTech (Wuhan, China). Antibody β-actin (sc-47778; 1:1,000) was obtained from Santa Cruz Biotechnology (Santa Cruz, CA, USA). Antibody D1 DA receptor (ADR001AN0302; 1:1,000) was purchased from alomone labs (Jerusalem Israel). Antibodies TH (ab137869; 1:1,000) and GABA_B_R (ab55051; 1:1,000) were purchased from Abcam (Cambridge, UK).

### Animals

Male C57BL/6 mice (22–25 g, 8 weeks) were obtained from Chongqing Medical University, China. Mice were housed in plastic cages under controlled condition: 12 h light/dark cycles, average temperature of 23°C, with free access to food and water. C57BL/6 human Trx-1 overexpression TG mice were constructed by (Cyagen Biosciences Inc., Guangzhou, China). The pronuclei of fertilized eggs from hyperovulated C57BL/6 were microinjected with human Trx-1 cDNA construct. The presence of Trx-1 transgene was confirmed by performing western blot and real-time PCR analysis (Figure S1 in Supplementary Material). Mice were divided into four groups: saline, morphine (20 mg/kg), TG + saline, and TG + morphine (*n* = 7 per group). All procedures and protocols were approved by the animal ethics council of Kunming University of Science and Technology and were in accordance with the National Institutes of Health Guide for the Care and Use of Animals (12). The lab procedures were also approved by the local Committee on Animal Use and Protection of Yunnan province (No. LA2008305).

### Western Blot Analysis

The VTA and NAc were dissected out according to the stereotaxic coordinates of Franklin and Paxinos, after the post-conditioning test. The exact coordinates for the two regions based on the center of the punch are: NAc (including core and shell): 1.34 mm anterior to bregma, 4.5 mm ventral to bregma, 0.8 mm lateral to the midline; and VTA: 3.4 mm posterior to bregma, 4.3 mm ventral to bregma, 0.5 mm lateral to the midline. After dissection, tissues were stored at −80°C until assay. Protein lysates was prepared using the solubilizing solution [1 mM EDTA, 20 mM Tris–HCl (pH 7.4), 1% NP-40, 150 mM NaCl, 1 mM phenylmethanesulfonyl fluoride, 1% Triton X-100, 1 mM EGTA, 2.5 mM sodium pyrophosphate, 1 mM β-glycerol phosphate, 1 mM Na_3_VO_4_, and 1 mg/ml leupeptin]. Protein concentration was determined using Bio-Rad protein assay reagent (Hercules, CA, USA). Using 12% (for GABA_B_R, D1, and TH) or 15% (for Trx-1) SDS-PAGE, equal quantity of proteins was separated and transferred to a polyvinylidene difluoride membrane (Millipore, Billerica, MA, USA). The membrane was soaked in 10% skimmed milk (in phosphate-buffered saline, containing 0.1% Tween 20, pH 7.2) for 2 h and incubated at 4°C overnight with the primary antibody. Immunoblots then were processed with the secondary antibodies (peroxidase-conjugated anti-mouse or anti-rabbit IgG) (1: 10,000, KPL, Gaithersburg, MD, USA). The bands were detected using an ECL chemiluminescence reagent kit (Millipore, MA, USA). Finally, densitometry analysis was performed by using ImageJ software.

### Conditioned Place Preference

**Figure 1:**
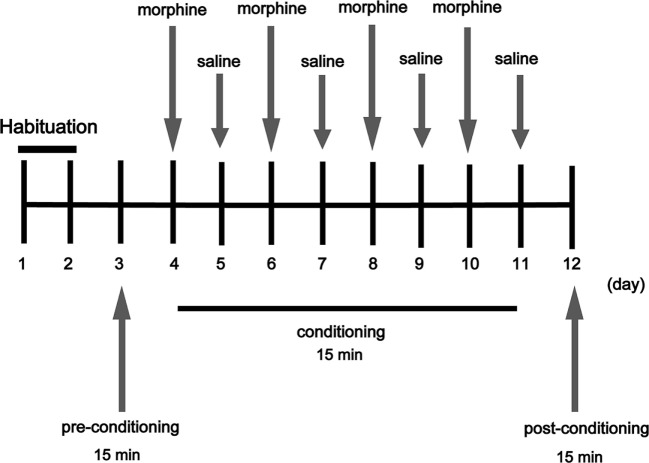
Experimental schedule for measurement of morphine-induced conditioned place preference in mice. Arrows indicate days on which behavioral tests were carried out (morphine 20 mg/kg).

The CPP apparatus (15 cm × 15 cm × 30 cm) consisted of two chambers divided by a Plexiglas sliding door, one chamber is a black wall and a rough floor, and the other one is a white wall and a smooth floor. Mice were given a 15 min pretest to verify that the box configuration did not produce a significant bias for either chamber. However, individual mice tended to spend more time in one chamber or the other during the pretest, thus mice were morphine paired in the chamber in which they spent the least amount of time during the pretest (13). The experimental schedule for the CPP task is shown in Figure 1; mice were given 2 days for habituation freely in the apparatus for 15 min/day. On day 3, mice were placed into the chamber and allowed to move freely between the white and the black chamber for 15 min for the pre-conditioning, record the time that the mice spent in each chamber to determine the preference of experimental mice before morphine administrator. On days 4, 6, 8, and 10, mice received a morphine injection (20 mg/kg) and were immediately placed into the appropriate chamber (morphine-paired chamber) of the CPP box for 15 min. Saline group and TG + saline group received a saline injection and were placed in the chamber (morphine paired) of the CPP apparatus for 15 min immediately. On days 5, 7, 9, and 11, mice received a saline injection and were immediately placed in the opposite chamber (saline-paired chamber) for 15 min. On day 12, the post-conditioning test was performed without drug treatment, and the time when the mice spent in each chamber was measured for 15 min and time spent in each chamber was evaluated to determine preference. The standard for us to determine whether mice were addictive (CPP) is that the mice which previously tended to prefer the dark chamber turned to tend to prefer the white chamber after morphine condition. According to the data for the pretest, we found that overall mice tended to prefer the dark chamber over the white chamber, thus most mice received morphine in the white chamber. The formula for calculation is as follows: Post–Pre value (s) = the time the mice spent in the white chamber in Post-CPP test − the time the mice spent in the white chamber in Pre-CPP test.

### High Performance Liquid Chromatography (HPLC) Analysis

The VTA and NAc samples (*n* = 6 per group) were ultrasonicated in 0.1 M perchloric acid containing 10 ng/mg of internal standard dihydroxybenzylamine. It was centrifuged at 12,000 rpm for 15 min. The concentrations of DA and GABA were measured by 1100 HPLC system equipped with an ECD-105 electrochemical detection (CoMetro) and XDB-C18 column (150 mm × 4.6 mm, 50 mM, Agilent Technologies). The mobile phase A for separation consisted of the following: Na_2_HPO_4_, 50 mM; trisodium citrate, 20 mM; triethylamine, 5 mM, pH 4.75; and l-heptanesulfonic acid 0.3 mM. The mobile phase B is methanol (A: B 1/4 95:5). The HPLC systems were controlled, and the data were collected by a computer equipped with ChemStation software from Agilent Technologies (14). The amounts of GABA and DA in each sample were calculated from calibration curves of standards which were run simultaneously with every set of unknown samples.

### Statistical Analysis

The data were expressed as mean ± SE values. Statistical analysis was performed using GraphPad Prism5 software. Normality was assessed using the Shapiro–Wilk test. A two-way ANOVA followed by a Bonferroni *post hoc* analysis was used to identify differences between treatment groups. A *P* value less than 0.05 was considered statistically significant.

## Results

### Overexpression of Trx-1 Blocked CPP Induced by Morphine

**Figure 2:**
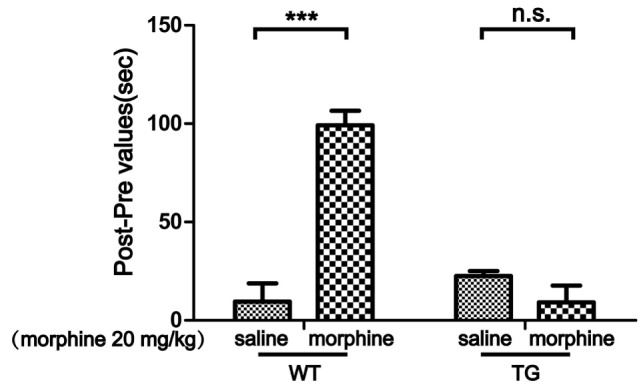
Effects of thioredoxin-1 (Trx-1) overexpression on morphine-induced conditioned place preference (CPP) in mice. The morphine-induced CPP was blocked in Trx-1 overexpressing transgenic (TG) mice. Mice were treated with morphine (20 mg/kg, intraperitoneally). Each bar represents the mean ± SE (*n* = 7). n.s. >0.05, ***P < 0.001, statistically significant.

Conditioned place preference is a model to examine the rewarding effects of drugs and other stimuli. The experimental schedule for the CPP is shown in Figure 1. The results showed that CPP was induced after morphine treatment in WT mice, while the CPP was not induced by morphine in Trx-1 TG mice (Figure 2). Two-way ANOVA revealed a significant mice × drug interaction (*F*_1,24_ = 48.33, *P* < 0.001) and significant effects of mice (*F*_1,24_ = 27.01, *P* < 0.001) and drug (*F*_1,24_ = 26.41, *P* < 0.001). Bonferroni *post hoc* test showed that significant difference between the saline and morphine group in WT mice (*P* < 0.001) but not in TG mice (*P* > 0.05).

### The Expression of Trx-1 in the VTA and NAc After Morphine-CPP Training

**Figure 3:**
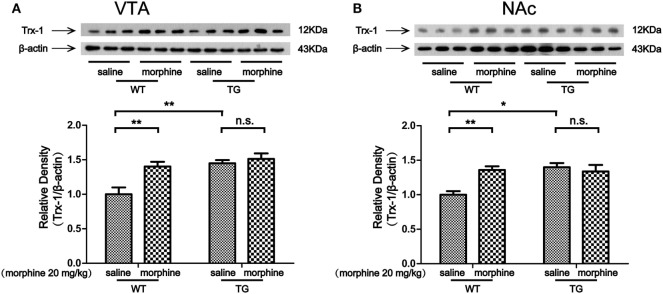
Thioredoxin-1 (Trx-1) expression in the ventral tegmental area (VTA) and nucleus accumbens (NAc). Immediately after the post-conditioning test, the VTA and NAc of mice were dissected out. Trx-1 expression was detected by western blot analysis. Trx-1 overexpression inhibited the further increase of Trx-1 by morphine in the VTA **(A)** and NAc **(B)**. Each bar represents the mean ± SE (*n* = 6). n.s. >0.05, *P < 0.05 and **P < 0.01, statistically significant.

The VTA and NAc are critically involved in CPP for both psychostimulants and opiates (15, 16). Our previous study showed that Trx-1 was induced by morphine *in vitro* and *in vivo* (9, 10). Thus, we first examined the expression of Trx-1 after morphine treatment. The expression of Trx-1 was induced by morphine in the VTA of WT mice, while the expression was not induced by morphine in TG mice (Figure 3A). Two-way ANOVA revealed a significant mice × drug interaction (*F*_1,20_ = 5.01, *P* < 0.05) and significant effects of mice (*F*_1,20_ = 13.62, *P* < 0.01) and drug (*F*_1,20_ = 9.36, *P* < 0.01). Bonferroni *post hoc* test showed a significant difference between the saline and morphine group in WT mice (*P* < 0.01) but not in TG mice (*P* > 0.05). The *post hoc* test also showed a significant difference between the TG and WT mice that have never been treated with morphine (*P* < 0.01). The expression of Trx-1 was induced by morphine in the NAc of WT mice; however, the expression of Trx-1 was not induced by morphine in TG mice (Figure 3B). Two-way ANOVA revealed a significant mice × drug interaction (*F*_1,20_ = 9.59, *P* < 0.01) and significant effects of mice (*F*_1,20_ = 7.82, *P* < 0.05) and drug (*F*_1,20_ = 4.85, *P* < 0.05). Bonferroni *post hoc* test showed a significant difference between the saline and morphine group in WT mice (*P* < 0.01) but not in TG mice (*P* > 0.05). The *post hoc* test also showed a significant difference between the TG and WT mice that have never been treated with morphine (*P* < 0.05).

### The Level of DA and the Expression of TH, D1 in the VTA and NAc After Morphine-CPP Training

**Figure 4:**
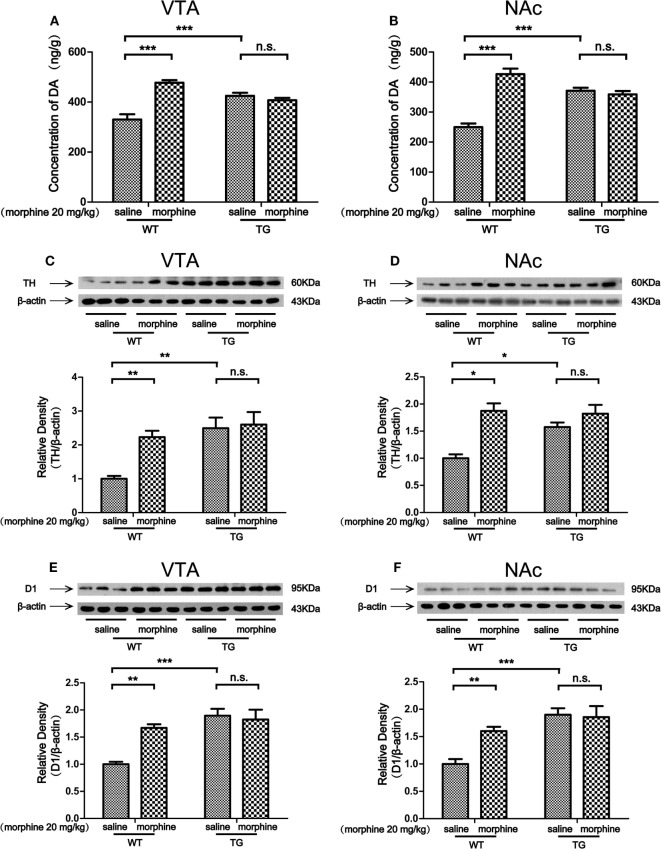
Dopamine (DA) concentration and the expressions of tyrosine hydroxylase (TH) and D1 in the ventral tegmental area (VTA) and nucleus accumbens (NAc). Immediately following the post-conditioning test, the VTA and NAc of mice were dissected out. DA concentration was detected by high performance liquid chromatography in the VTA **(A)** and NAc **(B)**. The expressions of TH and D1 were detected by western blot analysis. Thioredoxin-1 (Trx-1) overexpression inhibited the further increase of TH induced by morphine in the VTA **(C)** and NAc **(D)**. Trx-1 overexpression inhibited the further increase of D1 induced by morphine in the VTA **(E)** and NAc **(F)**. Each bar represents the mean ± SE (*n* = 6). n.s. >0.05, *P < 0.05, **P < 0.01, and ***P < 0.001, statistically significant.

Dopaminergic neurons in the VTA and NAc are activated in response to unpredicted rewards or cues that predict reward delivery (17). The DA receptor 1 (D1) is abundantly expressed in the VTA, especially on GABAergic neurons and synaptic afferents (18). Tyrosine hydroxylase (TH) is the maker of dopaminergic neurons (19). In this study, we found that the level of DA was increased by morphine in the VTA of WT mice, while the DA level was not increased by morphine in the VTA in TG mice (Figure 4A). Two-way ANOVA revealed a significant mice × drug interaction (*F*_1,20_ = 40.5, *P* < 0.001) and significant effects of mice (*F*_1,20_ = 5.61, *P* < 0.05) and drug (*F*_1,20_ = 32.06, *P* < 0.001). Bonferroni *post hoc* test showed a significant difference between the saline and morphine group in WT mice (*P* < 0.001) but not in TG mice (*P* > 0.05). The *post hoc* test also showed a significant difference between the TG and WT mice that have never been treated with morphine (*P* < 0.001). The level of DA was increased by morphine in the NAc in WT mice, while the DA level was not increased by morphine in TG mice (Figure 4B). Two-way ANOVA revealed a significant mice × drug interaction (*F*_1,20_ = 56.95, *P* < 0.001) and significant effects of mice (*F*_1,20_ = 6.33, *P* < 0.05) and drug (*F*_1,20_ = 31.10, *P* < 0.001). Bonferroni *post hoc* test showed a significant difference between the saline and morphine group in WT mice (*P* < 0.001) but not in TG mice (*P* > 0.05). The *post hoc* test also showed a significant difference between the TG and WT mice that have never been treated with morphine (*P* < 0.001). We found that the expression TH was increased by morphine in the VTA in WT mice, while the TH expression was not increased by morphine in TG mice (Figure 4C). Two-way ANOVA revealed a significant mice × drug interaction (*F*_1,20_ = 4.54, *P* < 0.05) and significant effects of mice (*F*_1,20_ = 12.48, *P* < 0.01) and drug (*F*_1,20_ = 6.46, *P* < 0.05). Bonferroni *post hoc* test showed a significant difference between the saline and morphine group in WT mice (*P* < 0.01) but not in TG mice (*P* > 0.05). The *post hoc* test also showed a significant difference between the TG and WT mice that have never been treated with morphine (*P* < 0.01). The results also showed that TH was increased by morphine in the NAc in WT mice, while the TH expression was not increased by morphine in TG mice (Figure 4D). Two-way ANOVA revealed a significant mice × drug interaction (*F*_1,20_ = 6.84, *P* < 0.05) and significant effects of mice (*F*_1,20_ = 4.83, *P* < 0.05) and drug (*F*_1,20_ = 21.81, *P* < 0.001). Bonferroni *post hoc* test showed significant differences between the saline and morphine group in WT mice (*P* < 0.05) but not in TG mice (*P* > 0.05). The *post hoc* test also showed significant differences between the TG and WT mice that have never been treated with morphine (*P* < 0.05). We found that the expression of D1 was increased by morphine in VTA in WT mice, while the expression of D1 was not increased by morphine in TG mice (Figure 4E). Two-way ANOVA revealed a significant mice × drug interaction (*F*_1,20_ = 10.08, *P* < 0.01) and significant effects of mice (*F*_1,20_ = 20.17, *P* < 0.001) and drug (*F*_1,20_ = 6.59, *P* < 0.05). Bonferroni *post hoc* test showed a significant difference between the saline and morphine group in WT mice (*P* < 0.01) but not in TG mice (*P* > 0.05). The *post hoc* test also showed significant differences between the TG and WT mice that have never been treated with morphine (*P* < 0.001). We found that the expression of D1 was increased by morphine in the NAc of WT mice, while the expression of D1 was not increased by morphine in TG mice (Figure 4F). Two-way ANOVA revealed a significant mice × drug interaction (*F*_1,20_ = 6.06, *P* < 0.05) and significant effects of mice (*F*_1,20_ = 19.36, *P* < 0.001) and drug (*F*_1,20_ = 4.54, *P* < 0.05). Bonferroni *post hoc* test showed a significant difference between the saline and morphine group in WT mice (*P* < 0.01) but not in TG mice (*P* > 0.05). The *post hoc* test also showed significant difference between the TG and WT mice that have never been treated with morphine (*P* < 0.001).

### The Level of GABA and the Expression of GABA_B_R in the VTA and NAc After Morphine-CPP Training

**Figure 5:**
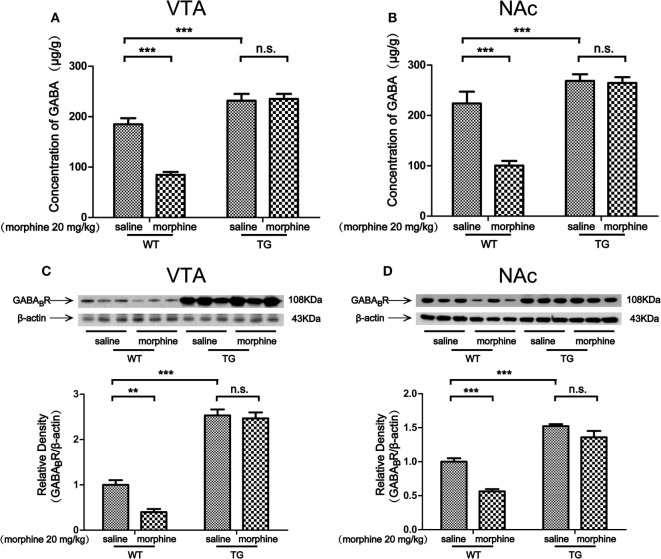
GABA concentration and the expression of GABA_B_R in the ventral tegmental area (VTA) and nucleus accumbens (NAc). After the post-conditioning test, the VTA and NAc of mice were dissected out. GABA concentration was detected by high performance liquid chromatography in the VTA **(A)** and NAc **(B)**. The expression of GABA_B_R was detected by western blot analysis. Thioredoxin-1 overexpression restored the expression of GABA_B_R suppressed by morphine in the VTA **(C)** and NAc **(D)**. Each bar represents the mean ± SE (*n* = 6). n.s. >0.05, **P < 0.01 and ***P < 0.001, statistically significant.

Morphine targets GABAergic interneurons in the VTA and NAc and decreases their activity, which leads to an indirect increase activity of dopaminergic neurons (2). We found that the level of GABA was decreased by morphine in VTA in WT mice, while the level of GABA was not decreased by morphine in TG mice (Figure 5A). Two-way ANOVA revealed a significant mice × drug interaction (*F*_1,20_ = 20.28, *P* < 0.001) and significant effects of mice (*F*_1,20_ = 73.71, *P* < 0.001) and drug (*F*_1,20_ = 17.64, *P* < 0.001). Bonferroni *post hoc* test showed a significant difference between the saline and morphine group in WT mice (*P* < 0.001) but not in TG mice (*P* > 0.05). The *post hoc* test also showed a significant difference between the TG and WT mice that have never been treated with morphine (*P* < 0.001). The level of GABA was decreased by morphine in the NAc of WT mice, while the level of GABA was not decreased by morphine in TG mice (Figure 5B). Two-way ANOVA revealed a significant mice × drug interaction (*F*_1,20_ = 16.11, *P* < 0.001) and significant effects of mice (*F*_1,20_ = 49.28, *P* < 0.001) and drug (*F*_1,20_ = 18.30, *P* < 0.001). Bonferroni *post hoc* test showed a significant difference between the saline and morphine group in WT mice (*P* < 0.001) but not in TG mice (*P* > 0.05). The *post hoc* test also showed a significant difference between the TG and WT mice that have never been treated with morphine (*P* < 0.001). We further detected the expression of GABA_B_R in the VTA and NAc. The expression of GABA_B_R was decreased by morphine in the VTA of WT mice, while the expression of GABA_B_R was not decreased by morphine in TG mice (Figure 5C). Two-way ANOVA revealed a significant mice × drug interaction (*F*_1,20_ = 5.68, *P* < 0.05) and significant effects of mice (*F*_1,20_ = 256.57, *P* < 0.001) and drug (*F*_1,20_ = 8.78, *P* < 0.001). Bonferroni *post hoc* test showed a significant difference between the saline and morphine group in WT mice (*P* < 0.01) but not in TG mice (*P* > 0.05). The *post hoc* test also showed a significant difference between the TG and WT mice that have never been treated with morphine (*P* < 0.01). The expression of GABA_B_R was decreased by morphine in the NAc in WT mice, while the expression of GABA_B_R was not decreased by morphine in TG mice (Figure 5D). Two-way ANOVA revealed a significant mouse × drug interaction (*F*_1,20_ = 5.51, *P* < 0.05) and significant effects of mice (*F*_1,20_ = 130.30, *P* < 0.001) and drug (*F*_1,20_ = 27.07, *P* < 0.001). Bonferroni *post hoc* test showed a significant difference between the saline and morphine group in WT mice (*P* < 0.01) but not in TG mice (*P* > 0.05). The *post hoc* test also showed a significant difference between the TG and WT mice that have never been treated with morphine (*P* < 0.001).

## Discussion

In this study, we found that overexpression of Trx-1 blocked morphine-induced CPP (Figure 2). Trx-1 expression was induced by morphine in the VTA and NAc. This result is consisted with our previous study. Trx-1 was induced by morphine in the VTA, NAc, and prefrontal cortex, as well as the inducer of Trx-1 protected mice from rewarding effects induced by morphine (10).

The VTA and NAc are critically involved in CPP for both psychostimulants and opiates (15, 20). Studies have shown that activation of dopaminergic neurons in the midbrain can induce place preference (21). Dopaminergic neurons in the VTA are predicted to play roles in rewarding effects. The VTA contains substantial heterogeneity in neurotransmitter type, dopaminergic and GABAergic neurons (22). The NAc is the main projection from the VTA (23). Thus, the increased expressions of TH and D1 induced by morphine in the VTA and NAc of WT mice were related to morphine-induced CPP. However, the level of DA as well as the expressions of TH and D1 was not induced by morphine in TG mice in which the CPP was blocked (Figures 4A–F). It has been reported that dopaminergic neurons in the VTA are inhibited by endogenous GABA (24). Morphine inhibits GABAergic neurons in the VTA and disinhibits dopaminergic neurons (25). GABAergic inhibition results in tonic and phasic ignition of dopaminergic neurons. Similarly, activation of GABA_B_R in the VTA by baclofen blocks both heroin self-administration behavior and DA release in the NAc (26, 27) and morphine-induced CPP (28). Taken together, GABA_B_R in the VTA may play an essential role in mediating opiate reinforcement and rewarding effects. The majority of neurons in the NAc are GABAergic. Thus, GABA_B_R in the NAc also plays an essential role in mediating opiate reinforcement and rewarding effects. The GABAergic neuronal inhibition in the NAc can be antagonized by elevating endogenous GABA concentration in the VTA. Our results showed that the GABA release and the GABA_B_R expression were decreased by morphine in the VTA and NAc of WT mice, while the alterations were inhibited in Trx-1 TG mice (Figures 5A–D). Thus, these results suggest that Trx-1 overexpression may block morphine-induced CPP through elevating endogenous GABA concentration and GABA_B_R expression.

Interestingly, the expressions of TH, D1, and GABA_B_R were upregulated by Trx-1 (Figures 4C–F and 5C,D). These results may be explained by the following studies. Our previous study showed that epinephrine increased TH expression through upregulating Trx-1 expression (14). Trx-1 increases the expressions of TH and D1 through increasing the expressions of c-fos and cAMP-response element binding protein (CREB) (29, 30). Forskolin enhances GABAergic responses (31). Trx-1 may increase GABA_B_R expression through upregulating forskolin/CREB. Thus, as our previous study suggested (11), an active defense system may have been built through increasing the levels of TH, D1, and GABA_B_R by the overexpression of Trx-1 in TG mice, which may contribute to the resistance of the development of CPP during morphine condition training.

In conclusion, Trx-1 overexpression blocks morphine-induced CPP in mice through regulating dopaminergic and GABAergic systems in the VTA and NAc. Our results indicate that Trx-1 may be a novel therapeutic target for morphine dependence.

## Ethics Statement

All applicable international, national, and/or institutional guidelines for the care and use of animals were followed. All procedures performed in studies were in accordance with the ethical standards of the institutional and/or national research committee and with the 1964 Helsinki declaration and its later amendments or comparable ethical standards.

## Author Contributions

JB was responsible for the study concept and design. XL, LG, XY, YL, XZ, and NG contributed to the acquisition of animal data. XL, MB, MH, and ZZ performed the western blot analysis. LY measured the concentrations of DA and GABA. XL and JB drafted the manuscript. JB and MH provided a critical revision of the manuscript for important intellectual content. All the authors read and approved the final version.

## Conflict of Interest Statement

The authors declare that the research was conducted in the absence of any commercial or financial relationships that could be construed as a potential conflict of interest.

## Supplementary Material

Figure S1 The expression of humanTrx-1 in the VTA and NAc.
