# Supplementary figures and images for: Overexpression of Thioredoxin-1 Blocks Morphine-Induced Conditioned Place Preference Through Regulating the Interaction of γ-Aminobutyric Acid and Dopamine Systems
Source: Front Neurol. 2018 May 2;9:309. doi: 10.3389/fneur.2018.00309 (PMC5941988; doi:10.3389/fneur.2018.00309)

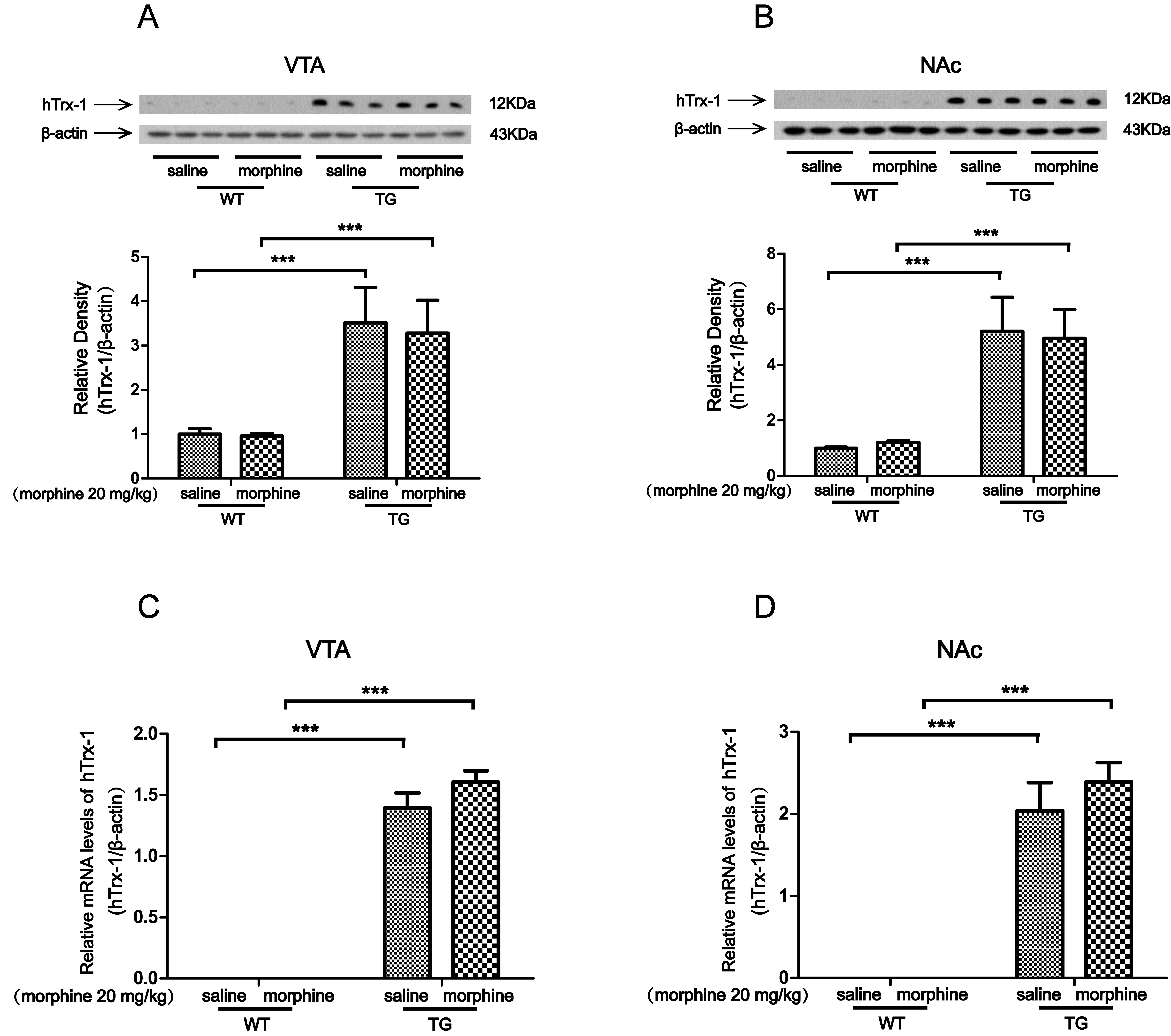

Supplement: Figure S1 — The expression of humanTrx-1 in the VTA and NAc. [file Image_1.tif]
