# Supplementary material for: Overexpression of Thioredoxin-1 Blocks Morphine-Induced Conditioned Place Preference Through Regulating the Interaction of γ-Aminobutyric Acid and Dopamine Systems
Source: Front Neurol. 2018 May 2;9:309. doi: 10.3389/fneur.2018.00309 (PMC5941988; doi:10.3389/fneur.2018.00309)
Supplement: Supplementary file 2 [file Presentation_1.PDF]

Overexpression of thioredoxin-1 blocks morphine induced conditioned place preference through regulating the interaction of γ-aminobutyric acid and dopamine systems

Xiang Li MS[1,2], Mengbing Huang MS[1,2], Lihua Yang MS[1,2], Ningning Guo MS[1,2] , Xiaoyan Yang MS[2], Zhimin Zhang MS[2], Ming Bai MS[2], Lu Ge MS[2], Xiaoshuang Zhou MS[2], Ye Li PhD[2], Jie Bai MD, PhD[2*]

1. Faculty of Environmental Science and Engineering, Kunming University of Science and Technology, Kunming 650500, China

2. Laboratory of molecular neurobiology, Medical Faculty, Kunming University of Science and Technology, Kunming 650500, China

** Corresponding author: Medical Faculty, Kunming University of Science and Technology, No.727 Jingming South Road, Kunming 650500, China*

Tel: +86-15025191617; Fax: +86-871-65920761; E-mail: jiebai662001@126.com

Supplementary information

Supplemental methods and materials

RNA extraction and Real-time PCR analysis

Total RNA was extracted from VTA or NAc tissue by using a Trizol reagent kit (CWBIO Corporation, Beijing, China) and converted to cDNA by using the Revert Aid TM First Strand cDNA Synthesis Kit (Fermentas, Walldorf Baden, Germany). The product was analyzed by using a Prism 7300 Sequence Detection System (Applied Biosystems, Foster, CA,USA). The following primer pairs were selected for real-time polymerase chain reaction (real-time PCR): gene expression was calculated relative to the housekeeping gene mouse β-actin. The primers as following: hTrx-1: F 5'-TTCCTTGAAGTAGACGTGGATGAC-3', R 5'-AGAGAACTCCCCAACCTTTTGAC-3', β-actin: F 5'-CAG TTC GCC ATG GAT GAC GAT-3', R 5' -ATC TGG GTC ATC TTT TCA CGG TTG-3'.

Reagents

Anti-human Trx-1 rabbit polyclonal antibody was owned by our laboratory.

Figure.S1 The expression of humanTrx-1 in the VTA and NAc

(A)The protein level of human Trx-1 in the VTA. Two-way ANOVA revealed no significant mice × drug interaction ($F_{1,20}$=0.03, P>0.05) and significant effects of mice ($F_{1,20}$=19.09, P<0.001) and no significant effects of drug ($F_{1,20}$=0.06, P>0.05). Bonferroni post hoc test showed no significant difference between the saline and morphine group in WT mice (P>0.05), and no significant difference in the VTA of TG mice (P>0.05). The post hoc test also showed significant difference between the TG and WT mice that have never been treated with morphine (P<0.001).

(B)The protein level of human Trx-1 in the NAc. Two-way ANOVA revealed no significant mice × drug interaction ($F_{1,20}$=0.08, P>0.05) and significant effects of mice ($F_{1,20}$=24.36, P<0.001) and no significant effects of drug ($F_{1,20}$=0.00, P>0.05). Bonferroni post hoc test showed no significant difference between the saline and morphine group in WT mice (P>0.05), and no significant difference in the NAc of TG mice (P>0.05). The post hoc test also showed significant difference between the TG and WT mice that have never been treated with morphine (P<0.001).

(C)The mRNA level of human Trx-1 in the VTA. Two-way ANOVA revealed no significant mice × drug interaction ($F_{1,20}$=1.91, P>0.05) and significant effects of mice ($F_{1,20}$=383.96, P<0.001) and no significant effects of drug ($F_{1,20}$=1.91, P>0.05). Bonferroni post hoc test showed no significant difference between the saline and morphine group in WT mice (P>0.05), and no significant difference in the VTA of TG mice (P>0.05). The post hoc test also showed significant difference between the TG and WT mice that have never been treated with morphine (P<0.001).

(D)The mRNA level of human Trx-1 in the NAc. Two-way ANOVA revealed no significant mice × drug interaction ($F_{1,20}$=0.71, P>0.05) and significant effects of

mice ($F_{1,20}=113.02$, $P<0.001$) and no significant effects of drug ($F_{1,20}=0.71$, $P>0.05$). Bonferroni post hoc test showed no significant difference between the saline and morphine group in WT mice ($P>0.05$), and no significant difference in the NAc of TG mice ($P>0.05$). The post hoc test also showed significant difference between the TG and WT mice that have never been treated with morphine ($P<0.001$).

Each bar represents the mean ± SE (n = 6). n.s. > 0.05, ***P < 0.001, statistically significant.
